# A data workflow to support plant breeding decisions from a terrestrial field-based high-throughput plant phenotyping system

**DOI:** 10.1186/s13007-020-00639-9

**Published:** 2020-07-16

**Authors:** Alison L. Thompson, Kelly R. Thorp, Matthew M. Conley, Michael Roybal, David Moller, Jacob C. Long

**Affiliations:** USDA-ARS Arid Land Agricultural Research Center, Maricopa, AZ 85138 USA

**Keywords:** Field-based high-throughput plant phenotyping, Database, Data processing, Plant breeding

## Abstract

Field-based high-throughput plant phenotyping (FB-HTPP) has been a primary focus for crop improvement to meet the demands of a growing population in a changing environment. Over the years, breeders, geneticists, physiologists, and agronomists have been able to improve the understanding between complex dynamic traits and plant response to changing environmental conditions using FB-HTPP. However, the volume, velocity, and variety of data captured by FB-HTPP can be problematic, requiring large data stores, databases, and computationally intensive data processing pipelines. To be fully effective, FB-HTTP data workflows including applications for database implementation, data processing, and data interpretation must be developed and optimized. At the US Arid Land Agricultural Center in Maricopa Arizona, USA a data workflow was developed for a terrestrial FB-HTPP platform that utilized a custom Python application and a PostgreSQL database. The workflow developed for the HTPP platform enables users to capture and organize data and verify data quality before statistical analysis. The data from this platform and workflow were used to identify plant lodging and heat tolerance, enhancing genetic gain by improving selection accuracy in an upland cotton breeding program. An advantage of this platform and workflow was the increased amount of data collected throughout the season, while a main limitation was the start-up cost.

## Background

Field-based high-throughput plant phenotyping (FB-HTPP) research programs have steadily increased over the last 10 years. Defined as the ability to rapidly and accurately phenotype large numbers of field grown plants, FB-HTPP has been a primary focus for crop improvement to meet the demands of a growing population in a changing environment [1–3]. A widely held understanding is that novel breeding strategies which exploit the natural, genetic variation of high-production and high-quality agricultural crops will be a sustainable approach to improve yield [4, 5]. One of the biggest bottlenecks in plant breeding today is the ability to rapidly phenotype large populations throughout the growing season. Current manual techniques are labor intensive and time consuming, and often introduce unwanted variation in the collected data [1]. FB-HTPP when applied to breeding programs can contribute toward improving selection intensity with larger field trials, increasing selection accuracy by reducing human error, and identifying novel genetic variation by capturing multiple phenotypes over time [6].

Many of the early FB-HTPP adopters adapted techniques from aerial and satellite-based remote sensing [7] to close-range, “proximal” deployment of sensors and imagers on terrestrial vehicles [3, 8–14]. Since then unmanned aerial systems (UAS) and field-scanners have been increasing in popularity. With these various systems, breeders, geneticists, physiologists, and agronomists have been able to improve the understanding between complex dynamic traits and plant responses to changing environmental conditions. Using terrestrial-based platforms, Pauli et al. [15] identified temporal patterns of quantitative trait loci (QTL) expression for measured canopy temperature in an upland cotton recombinant inbred line population, while Tanger et al. [16] identified four alleles that had a negative impact on grain yield in rice. Rutkoski et al. [17] improved the accuracy of genomic selection models in wheat using UAS derived traits. Ge et al. [18] characterized temporal dynamics in plant growth, including water use, using hyperspectral and red, green, blue imagery, while Thorp et al. [19] identified cotton varieties with improved water use efficiency from UAS derived fractional vegetation cover using multispectral images. Chlorophyll fluorescence imaging has been used to track the effects of viral and fungal pathogens, showing a reduction in photosynthesis, before visible symptoms occurred [20, 21].

While HTPP has great potential for plant breeding, the volume, velocity, and variety of data captured can be problematic, requiring large data stores, databases, and computationally intensive data processing pipelines before application to quantitative trait loci mapping, genome wide association studies, genomic selection, and other statistical analyses. For FB-HTPP to be useful for plant breeders, novel workflows including applications for database implementations, data processing, and data interpretation must be developed and optimized [22]. To gain the most impact from HTPP data, analysis should occur in near real-time to (1) check for errors in the data due to sensor or logger malfunctions and (2) guide field management or future collection decisions [23]. Fiorani and Schurr [24] further stipulated that these data management schema integrate experimental metadata to link the measured phenotypes with the environmental conditions to enable analysis of phenotypic responses.

The main objective of this research was to build upon experience with prior platforms and workflows and incorporate novel components for data management and analysis from a sensing system on a high-clearance tractor, used primarily to support cotton breeding objectives at the USDA-ARS research station in Maricopa, Arizona. Specific objectives were to (1) describe each aspect of the workflow, including data collection, database design, geospatial processing, quality control, visualization, and outlier removal and (2) demonstrate the value of the methodology as applied to the cotton breeding program at Maricopa, Arizona.

## Field-based high-throughput phenotyping workflow

### Overview

**Fig. 1:**
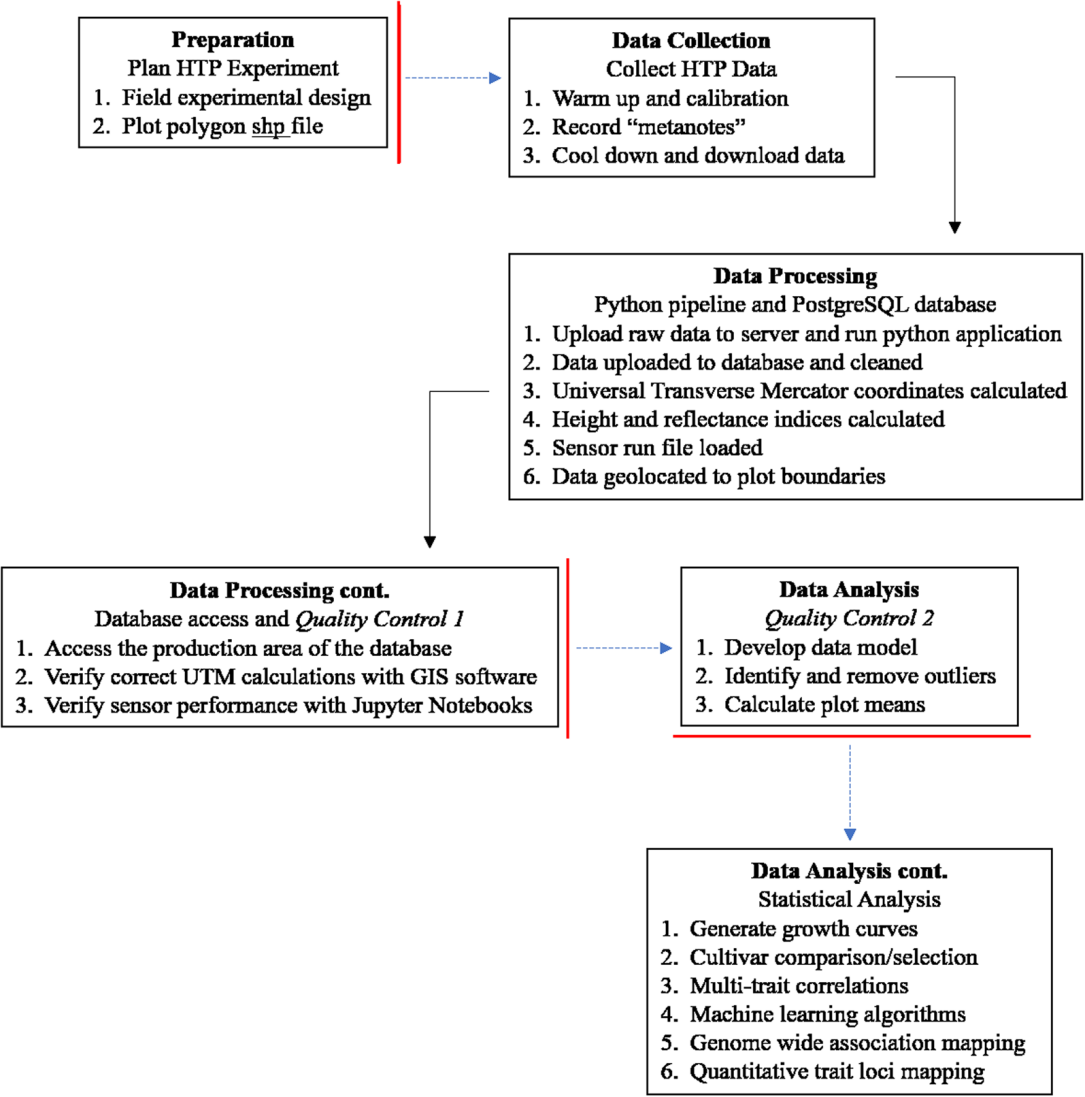
A workflow for the high-clearance tractor sensor system. Each component contains the work conducted in that section. The red bars and blue dotted arrows indicate user input is required before continuing, the black arrows indicate no user input is required to move on to the next component

A general workflow including four main components for FB-HTPP including (1) Preparation, (2) Data collection, (3) Data processing, and (4) Data analysis was described by Thompson el al [14] and were applied to the sensing system (Fig. 1). The Preparation and Data collection components for the system differ very little from Thompson et al. [14] so will not be covered in detail. In the Data processing component, data are uploaded to a PostgreSQL database via a Python GUI application, and data are georeferenced and spatially linked to experimental plots. In the Data analysis component, 2 quality control steps are performed before subsequent statistical analysis to make decisions for the breeding program.

### High-clearance platform and sensor package

An Avenger-Pro high-clearance tractor (LeeAgra Inc., Lubbock, TX, USA) was identified as the terrestrial vehicle for retrofitting a FB-HTPP sensing package and data acquisition system. Two features of this platform were critical for local FB-HTPP activities. First, the tire spacing was adjustable via a hydraulic system to accommodate plant row spacings, ranging from 1.80 to 3.07 m. Second, hydraulic lift systems were available to vertically elevate both the vehicle platform and the sensor boom to accommodate variable plant heights over time, with a total range of 1.04–2.74 m. The front boom was modified by adding a custom frame constructed from 0.04× 0.04 m extruded aluminum T-slot tubing, framing members, and hardware (Rexroth Bosch Group, Charlotte, NC, USA) for attaching proximal sensors and associated hardware as previously described by Thompson et al. [25].

Since 2014, several different sensors have been added to and removed from the Avenger sensor array; however, this workflow will focus on the core, “tri-metric” sensor package which includes Pepperl + Fuchs UC2000 (Pepperl + Fuchs Group, Twinsburg, OH, USA) ultrasonic transducers to measure canopy height, Apogee SI-131 (Apogee Instruments, Logon UT, USA) infrared thermometers (IRT) to measure canopy temperature, and Crop Circle ACS-470 (Holland Scientific, Lincoln, NE, USA) active spectral reflectance sensors to measure normalized difference vegetative index (NDVI). Measurements from each sensor were georeferenced by simultaneously recording (1) vehicle position from a Trimble R6 real-time kinematic (RTK) GPS receiver (Trimble Inc., Sunnyvale, CA, USA) via the GGA and RMC NMEA strings and (2) vehicle heading from an inertial measurement unit/attitude and head reference system (IMU/AHRS) sensor (VN-100, VectorNav Technologies, LLC, Dallas, TX). Sensor offset distances from the RTK-GPS receiver were measured and recorded for geospatial processing, as described below. Table 1 provides information about each sensor, and the positions of each sensor on the boom are shown in Additional file 1: Figure S1. Other sensors were mounted to the sensor boom but will not be discussed in this paper.

**Table 1:** List of model name, manufacturer, approximate price, number of units purchased, equipment purpose, logging system, and communication (Comm) method for each sensor used on the Avenger high-throughput phenotyping platform

Figure no	Equipment	Manufacturer	Cost (USD)	Units	Total cost (USD)	Purpose/trait captured	Logger system	Comm method
1	Trimble R6 receiver	Trimble Inc	$6995	1	$6995	GPS coordinates	PXI-e	RS232
2	PXI-e 1085	National Instruments	$58,000	1	$58,000	Data logger	n/a	n/a
3	VN-100	VectorNav Technologies	$1250	1	$1250	Inertial measurement and heading	PXI-e	RS232
4	Apogee SI-131	Apogee Instruments	$682	8	$5456	Canopy temperature	PXI-e	Voltage
5	Crop Circle ACS-470	Holland Scientific	$5000	8	$40,000	Active spectral reflectance	PXI-e	RS232
6	Pepperl + Fuchs UC2000	Pepperl + Fuchs Group	$362	4	$1448	Plant height	PXI-e	RS232
7	HC2S3 probe	Campbell Scientific	$294	1	$294	Ambient temperature/relative humidity	CR1000	Voltage
8	CR1000	Campbell Scientific	$2186	1	$2186	Data logger	n/a	n/a
9	Apogee SP-110	Apogee Instruments	$217	1	$217	Solar irradiance	CR1000	Voltage
10	Dynasys auxiliary power	Tridako Energy Systems	$9600	1	$9600	Power	n/a	n/a

These equipment prices reflect special quote pricing when available and some equipment models may no longer be available. Total cost for the equipment was $125,446

### Sensor data logging and power

Power to the sensors and data loggers was supplied by a retrofitted 110 V AC Dynasys auxiliary power unit (Tridako Energy Systems Inc., Alliance, NE, USA) sourced at 12 and 24 V. Sensor information was logged on a PXI-e 1085 data acquisition system (National Instruments, Austin, TX, USA). The PXI-e used a Windows 7 operating system, and LabView 2014 code (National Instruments, Austin, TX, USA) was developed for data acquisition. A discrete 200 ms loop was used to sample sensor measurements and write sensor data to text files with an accompanying timestamp. The sensor output signals were either an analog voltage or current, or a serial RS232 communication updating at a rate of 5 Hz. The analog signals were recorded as 0–5 V potentials, and the RS232 communication signals were decoded to ASCII text. The communication method for each sensor is indicated in Table 1.

### Data collection protocol

On scheduled collection days, the sensor platform and data acquisition system aboard the Avenger tractor underwent a warm-up and calibration period at least one hour prior to the start of the field collection. If any sensor readings fell outside a pre-determined optimal range, they were re-calibrated or replaced as appropriate. A 24-step check list for the start-up and warm-up procedure for the Avenger platform is provided in Additional file 5: Table S1. After the warm-up period was complete the platform height and wheel spacing was adjusted to fit the field design and crop for collection. Metadata on all activities were recorded in a notebook, then later transcribed to an electronic document (Additional file 2: Figure S2). During data collection, the tractor’s forward speed was maintained at 0.67 ms^−1^. Once the collection was complete, the Avenger tractor underwent a cool-down period. The warm-up and cool-down periods were critical to identify any sensor malfunctions that may have occurred during the collection and provide points of reference to correct data when possible. After the cool-down period, data from the internal 5-RAID hard drive of the PXI-e was transferred to an external solid state USB3 drive. The files were transferred to a windows server following specific directory and file naming conventions utilized by the processing pipeline application described below. The directory and file naming conventions were retained from the prior workflow described by Thompson et al. [14]. Users found the conventions straight forward and easy to follow and, by keeping the same structure, provides users the opportunity to use the new processing pipeline, database, and quality control steps described below on archived data collections.

### Data storage and hardware

The data collected with the Avenger platform was stored on a Dual Intel Xeon 12-Core processor server with Windows Server 2012Rs operating system and additional JBODs (Just a Bunch of Drives). The first JBOD contained 24, 8 TB Enterprise HGST 12 Gb/s 7.2 K RPM drives (Western Digital, San Jose, CA, USA) and was directly connected to the ARECA RAID card on the server via mini-SAS3 cables. The second JBOD contained 44 drives and was connected to the first JBOD out port in a daisy-chain manner. The server had 512 GB of memory, 500 TB of storage post-formatting, and was equipped with a 5000 core GPU Nvidia K80 Tesla card for graphics but was not used by the application described below. The HTP server was integrated with the USDA network and local area network at Maricopa. All users of the HTP server have read permissions (i.e., can access, view, and copy files from the server) but only the core HTP team has read/write permissions (i.e., can create or delete folders and files, as well as access, view, and copy data). This ensures raw HTP data is properly formatted, maintained, and secured and that the server’s file system is efficiently maintained. The server was backed up to tape (15 TB LTO-7 Ultrium, Hewlett Packard Enterprise, San Jose, CA, USA) using a HP MSL2024-LT07 dual tape drive (Hewlett Packard Enterprise, San Jose, CA, USA) following a two-part back-up schema. The first back-up was iterative and occurred every Friday (i.e., only new data for that week is backed up); the second back-up was the full server and occurred the last Saturday of every month. ArcServe 18.0 software (Arcserve, Eden Prairie, MN, USA) was used to schedule and manage tape back-ups. The full server back-up typically required 20–25 h to complete onto 3 tapes.

### Database design and structure

The HTP server also incorporated the Python graphical user interface (GUI) application and PostgreSQL (www.postgresql.org) relational database for the Avenger platform. The database software chosen was PostgreSQL 9.5, because it met four criteria of the ALARC Plant Phenotyping group; (1) the software must be open source and low cost; (2) it must work with multiple operating systems; (3) it must be able to support large quantities of data and multiple concurrent users; and (4) it must support transactions where either all data is submitted to the database or none. These criteria ensured that many colleagues and collaborators had access to the database and that partial datasets did not contaminate analysis. These steps were critical for long-term management of these datasets.

**Fig. 2:**
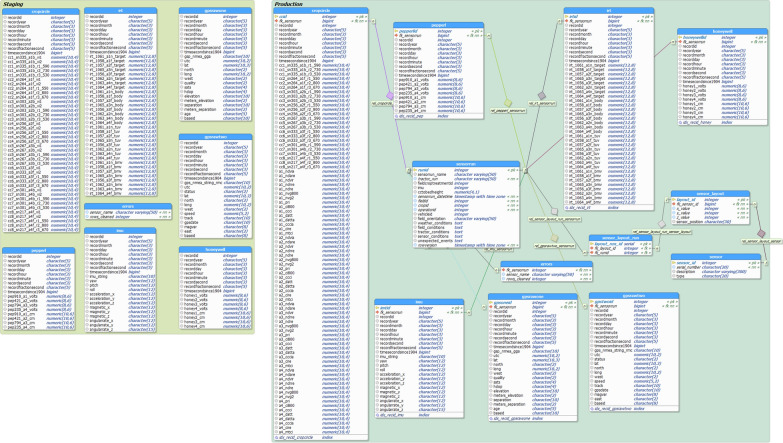
An entity relationship diagram of the PostgreSQL relational database with the different areas (production, staging, public) designated. Physical tables are denoted with a blue header and Views (virtual tables) are denoted by a yellow header

The programming language used for the database and its associated GUI application was Python 2.7. The database design was split by function into three areas: Staging, Production, and Public (Fig. 2). The first step to begin data upload into the database was to create a sensor run file using the metadata recorded during the Avenger collection. This file was needed by the database to create a master record that related all the output files from a collection or “run”. After running the application, the first step was to navigate to the folder containing the sensor run file and raw data from the PXI-e. Each file was then parsed (cleaned) so that bad characters, or incomplete data lines, were not brought into the database, while keeping the raw files unmodified. The parsed files were then read and inserted into Staging tables in the database. The application then outputted the parsed or “clean” files back into the parent directory in a new folder (clean).

After the files were created and cleaned, the Python application made several initial calculations and adjustments to the data, including georeferencing, plant height calculations, and spectral reflectance indices. First, the latitude and longitude coordinates from the RTK-GPS receiver position at each logger timestamp were projected to the Universal Transverse Mercator (UTM) coordinate system (units of meters) as previously described by Wang et al. [26]. The UTM position of each sensor was then calculated from the vehicle position, IMU heading data, and the known lateral and forward offset distances of each sensor to the RTK-GPS receiver. The final calculations were the adjusted plant height and the reflectance indices (NDVI, NDRE). The adjusted plant height (*h*) was calculated as:

$$h = s - d$$

where s is the recorded soil line to sensor boom height and d is the displacement data measured by the ultrasonic sensors. The reflectance indices were calculated as:

$$NDVI =\frac{\rho NIR-\rho VIS}{\rho NIR+\rho VIS}$$

$$NDRE =\frac{\rho NIR-\rho RE}{\rho NIR+\rho RE}$$

where *ρNIR* is reflectance at 800 ± 5 nm, *ρVIS* is reflectance at 670 ± 5 nm, and *ρRE* is reflectance at 730 ± 5 nm respectively.

Once the initial calculations and adjustments were complete, the values (UTM coordinates, sensor UTM coordinates, NDVI) were appended to the parsed Staging tables and the sensor run text file was read and loaded as a completed run into the ‘sensorrun’ table. This created a permanent record of the collection in the database. A stored procedure in the database then moved the data from the Staging tables into the Production area tables and associated them with the ‘sensorrun’ table entry that contains the metadata. This step linked all the data from the Staging tables to the Metadata for that collection. This step is very important as the captured metadata enables users to link other, non-HTP derived experimental and management data to the database. Next, the Python application generated comma separated files and wrote them back into the parent directory in a new directory (processed). These output files came from views within the database. A view is a virtual table that can combine information from two or more tables. For each of the sensor types, the files included the sensor ID, UTM coordinates, timestamp, corresponding data values, and other calculated values when applicable. If a geospatial file designating experimental plot boundaries in UTM coordinates was provided, new files containing data within those boundaries were outputted to the parent directory in a new folder (clipped). Wang et al. [26] described several methodologies for creating georeferenced maps that delineate plot boundaries, including using a geographic information system (GIS) to design field plot maps in shapefile format and an algorithm that calculated plot boundaries from georeferenced sensor data. Due to local availability of GIS expertise and RTK-GPS equipment, the Maricopa HTPP program typically obtains plot boundary maps using the former approach. These output files contain the same information kept in the Production tables of the database. After the Production tables were generated and comma separated files outputted, the Staging tables were deleted and ready for the next collection to be loaded.

### Accessing the database

**Fig. 3:**
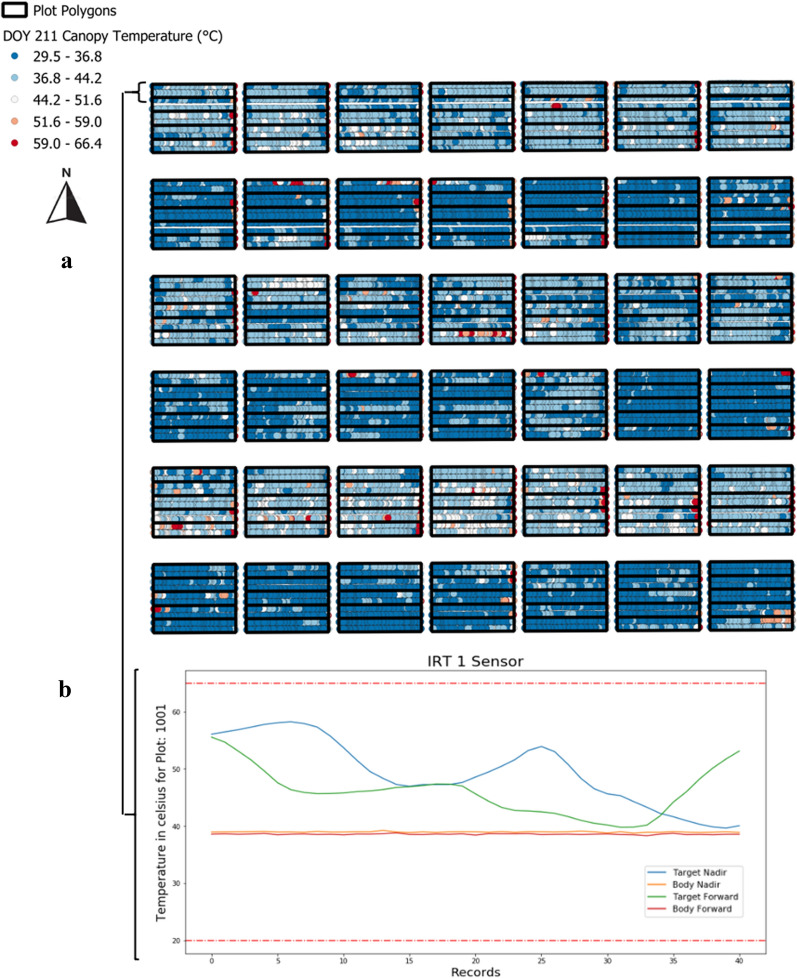
Infrared thermometry data (°C) for DOY 211 (30-Jul-2015) visualized within the geospatial plot boundaries using QGIS software. The image shows the well-watered treatments with cooler canopy temperatures and the water-limited treatments with increased canopy temperatures. This provides visual confirmation to the user that irrigation treatments are having an effect (**a**). Infrared thermometry data for a single plot (1001) visualized within the Jupyter Notebook using the “matplotlib” package. The pre-determined canopy temperature boundaries are the red dashed lines. The canopy temperature within the geospatial plot boundaries are the blue and green lines while the solid red and orange lines are the body temperature of the sensor. This provides visual confirmation to the user that the sensors are working correctly and returning reasonable canopy temperature values for cotton grown in Arizona (**b**)

The database created for the Avenger data is a geospatially enabled relational database and meets the American National Standard Institute (ANSI) standards for Structured Query Language. To retrieve data or subsets of data (custom reports), the user must understand Structured Query Language (SQL). Data can be accessed in the Public area by any application that can access a database, such as geographic information system (GIS) software, with a user ID and password. Both ArcGIS (ESRI, Redlands, CA, USA) and Quantum GIS (QGIS, www.qgis.org) can access the Avenger PostgreSQL database. Tutorials for both ArcGIS and QGIS on establishing database connectivity to a PostgreSQL database are available online. In QGIS, the PostGIS plugin enables users to develop SQL statements via the query builder application. An advantage to establishing database connectivity to a GIS program is that users can quickly see if all UTM conversion, sensor offset, and plot boundary calculations were performed correctly within minutes of being uploaded to the database. The GIS software also enables users to quickly visualize experimental treatment or genotype differences captured by the data (Fig. 3a). This near real-time visualization can guide field management and/or future data collection decisions, a data pipeline criterion established by White et al. [23] for FB-HTPP data. Other software programs such as SAS (SAS Institute, Cary, NC, USA), Jupyter Notebook (www.jupyter.org), R Studio (R Studio, Boston, MA, USA), and Matlab (MathWorks Inc., Natick, MA, USA) can also access the database and can be used to develop custom data analysis and processing applications.

### Data quality control: Step 1

Before statistical analysis of the experimental parameters (i.e. treatment or genotype effects) can be assessed for the Avenger collected data, a quality control (QC) step must be performed. This step, which was missing from the prior Thompson et al. [14] workflow, is crucial to identify any logging or sensing errors that might have been missed during the warm-up and cool-down process. The QC pipeline was written in Python 2.7 and utilizes Jupyter Notebook for the user interface. A separate notebook was developed for each sensor type (Crop Circle, IRT, ultrasonic) to easily visualize common errors that can occur. The notebooks utilize “psycopg2”, a PostgreSQL database adapter for the Python programming language, to access the Public area of the database. The “ipywidgets” package was utilized to develop drop-down options within the notebooks to make navigation of the custom reports (SQL statements) more user friendly. The “matplotlib” package was used to generate graphs for data visualization. The QC notebooks provided simple statistics about each run and compared those statistics to previous runs stored within the database. This enables users to see any gradual degradation of sensor outputs that might otherwise be overlooked when focused on a single collection. The notebooks also allow users to see that the same number of records are being collected for each sensor on each arm of the sensor boom. Differences in the records would alert users to potential problems in the data logging itself (i.e. data loops were not being returned after the designated amount of time). Users can visualize the data against pre-determined thresholds (e.g. sensor operational ranges or known “impossible” data outputs) for quick assessment of the data quality (Fig. 3b). If the data quality does not meet user standards, the sensors can be inspected, and a new collection can be taken. It is also possible for users to attempt data corrective actions using the warm-up and cool-down values if a pattern of error can be detected.

### Data quality control: Step 2

**Fig. 4:**
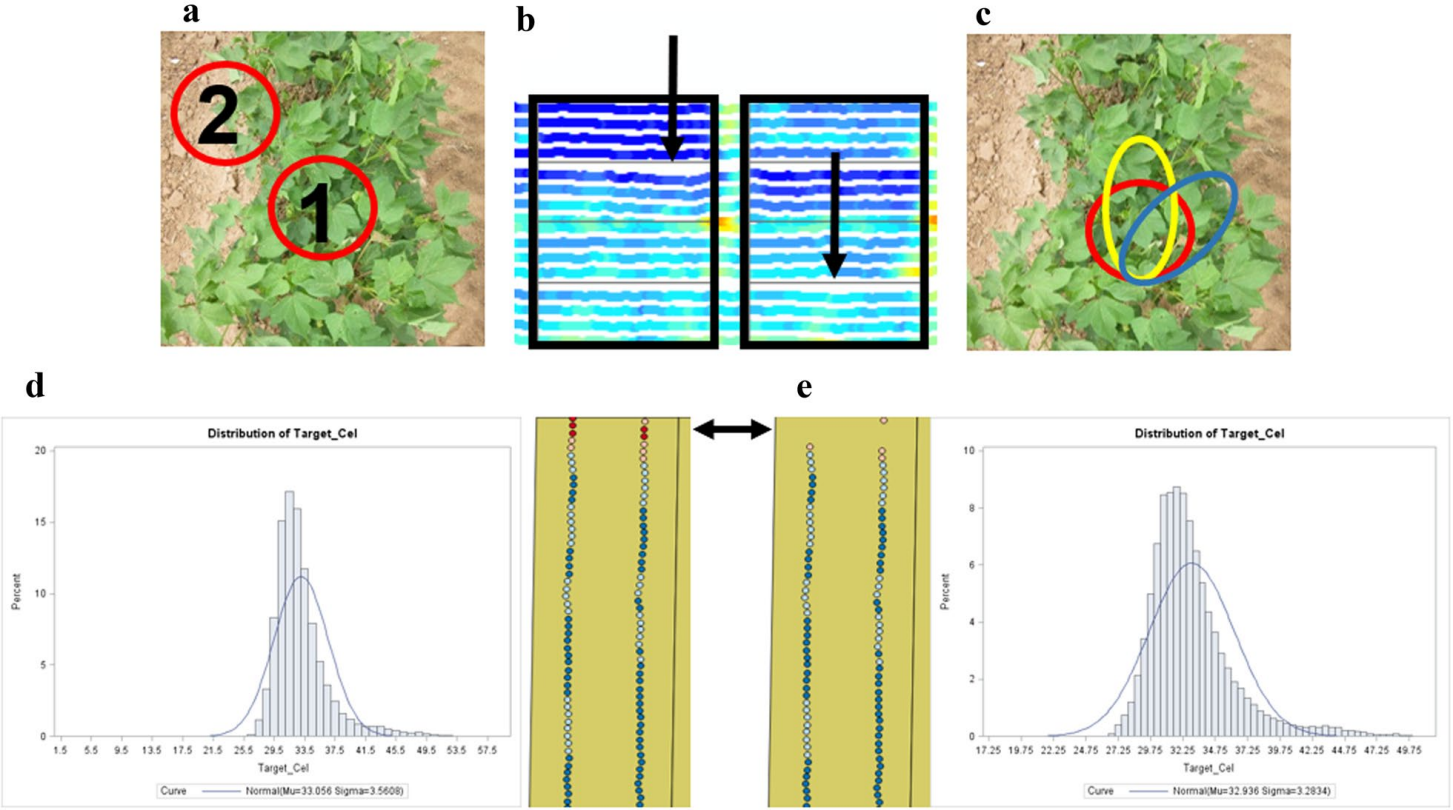
Image of cotton plants with the infrared thermometer (IRT) field of view (FOV) outlined in red (**a**), where FOV1 is centered on the cotton crop and FOV2 is no longer centered on the cotton crop due to a row centerline deviation (wobble). The “wobble” visualized in QGIS software within the experimental plot boundaries (**b**). Changes in the sensor FOV due to sensor boom roll and pitch alterations because of holes and bumps in the tractor drive path (**c**). An example of pre-outlier (**d**) removed data where the distribution is highly variable, and outliers caused by edge effects are visible in the north end of the plot. An example of post-outlier (**e**) removed data from an infrared thermometer after a single iteration where the edge effect outliers are removed, and the data better resemble a normal distribution (blue line)

Once the data QC1 outputs were complete and inspected, an outlier removal process was implemented (QC2). Even though the data was checked for sensor and logging errors, data points may still differ significantly from others within the experimental plot boundaries. Two main sources for outliers have been identified and are removed in the steps described below. These are (1) sensor position deviation from the crop row centerline while data is collected due to error in maneuvering the Avenger platform (Fig. 4a, b), dubbed a “wobble”, and (2) plant sparsity which can occur because of poor germination, edge effects, or poor management practices. In either situation, there is not enough plant material in the sensor field of view for a representative value and instead soil or field debris is incorporated into the measurement. Another source for outliers comes from inevitably driving the platform over large holes or bumps (i.e. clods) in the field, which changes the pitch and roll of the sensor boom and alters the effective field of fiew of the sensors (Fig. 4c). The latter type of outliers can be more difficult to identify and remove from the dataset; however, the yaw, pitch, and roll values outputted from the VectorNav IMU/AHRS can be used to set a threshold for data removal, if desired, or quantify the physcial dynamics of the data. As with all processing for *Data quality control: Step 1*, users must determine what is appropriate for their experimental design and objectives.

To remove outliers, the HPMIXED procedure within SAS (SAS Institute, Cary, NC, USA) was used to fit a mixed linear model to each trait (height, canopy temperature, reflectance indices). Outliers were determined by examining the Studentized deleted residuals obtained from the model [27]. The model parameters depended on the experimental design and project objectives. Examples of models to remove outliers from field-based HTP collections can be found in Andrade-Sanchez et al. [3], Pauli et al. [15], and Thompson et al. [14, 25]. Outliers are removed from the data in an iterative fashion (Fig. 4d, e). Once outliers are removed, plot-level means can be calculated using the MEANS procedure within SAS, or users can perform other types of analyses. Users can also utilize custom scripts in R or Python to remove outliers.

## Application to plant breeding

**Fig. 5:**
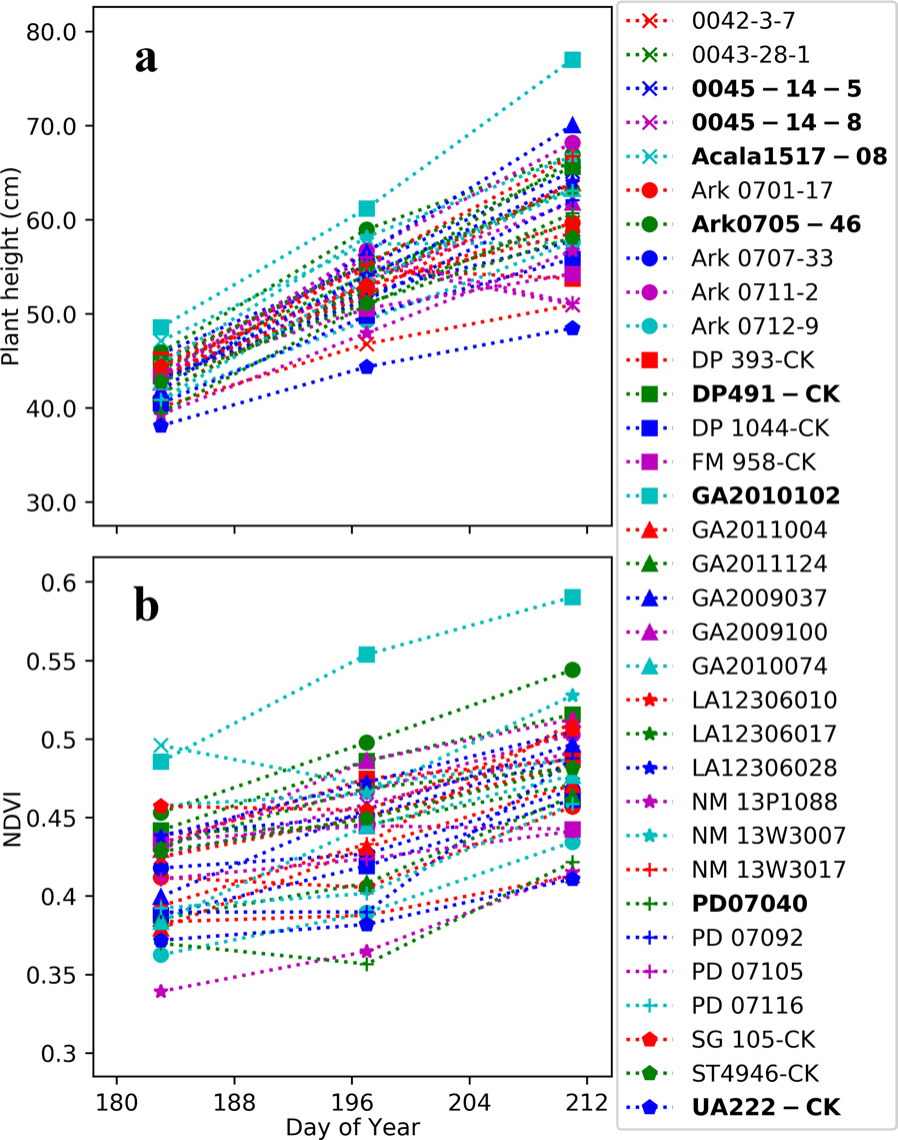
The calculated plant height (**a**) and normalized difference vegetative index (NDVI) (**b**) of thirty-three irrigated upland cotton breeding lines and variety checks as collected by the Avenger tractor on 02-July (DOY183), 16-July (DOY197), and 30-July (DOY211) in 2015 at the U. of Arizona Maricopa Agricultural Farm in Maricopa Arizona, USA. The graphs were generated with the “matplotlib” package for Python v3.0

As with traditional field measurements, the data from the Avenger tractor can be used to select for or against certain germplasm based on simple agronomic characteristics. For example, final plant height (Fig. 5a) indicates line GA2010102 would not be a good commercial line for the Maricopa location, because it grows taller than the cotton picker header which can cause the main stem to break and introduce trash during mechanical harvest. Plotting plant height over time can identify germplasm prone to lodging due to storms. The lines 0045-14-5, 0045-14-8, and Acala 1517-08 reduced in height by almost 10 cm between DOY197 and DOY212. During this time frame, monsoon storms (high wind and heavy rain) are common and can cause cotton varieties with weak stems to split and fall over. Using this data, a breeder would determine these lines to be storm intolerant and would likely discontinue them from the breeding program.

Plotting the normalized difference vegetative index (NDVI) over time (Fig. 5b) shows lines adversely affected by the onset of high temperatures typical in Arizona during July and August. Several lines including Acala 1517-08, PD07040, and the DP 491 check variety stagnate in growth with the onset of the elevated temperatures. Once the period of stagnation is over the growth rate is less than what is was before the elevated temperatures began indicating these lines are susceptible to heat stress. However, lines GA2010102, Ark 0705-46, and the UA 222 check variety maintain steady growth even after the onset of heat indicating they are well adapted for heat stress. With this information breeders and physiologists can develop future experiments to verify the heat adaptation and identify physiological mechanisms for adaptation. Breeders can also develop mapping populations to identity genetic regions and molecular markers associated with heat adaptation to improve breeding efficiency with marker-assisted or genomic selection.

**Fig. 6:**
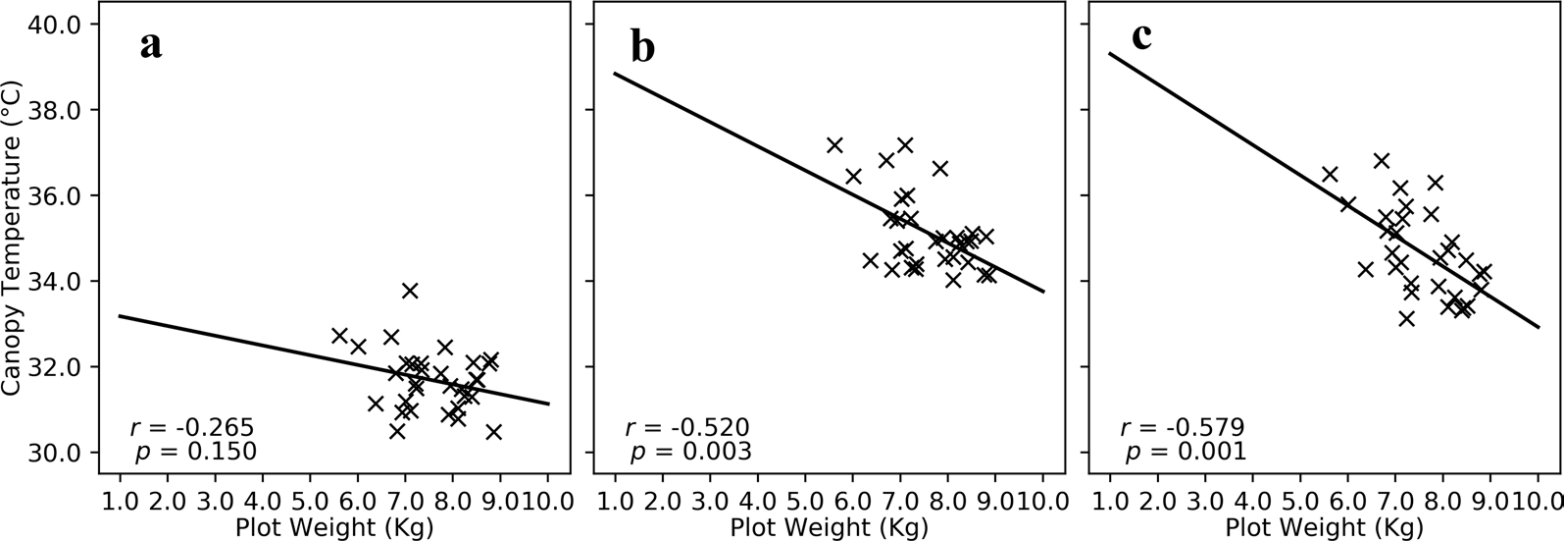
Linear regression of canopy temperature to plot weight (yield) under water-limited conditions for (**a**) canopy temperature measured 02-July (DOY183), (**b**) canopy temperature measured on 16-July (DOY197), and (**c**) canopy temperature measured on 30-July (DOY211) in 2015 at the U. of Arizona Maricopa Agricultural Center in Maricopa Arizona, USA

The data provided by the Avenger platform can also be used to drive novel selection criteria and predict yield parameters to improve the effectiveness of a breeding program. For example, reduced canopy temperature has often been associated with increased yields in a variety of crops [28–31]. Breeders may select lines based on this criterion in early-generation field trials where small plots make accurate yield estimates difficult. Depending on the number of plots in the trials, a breeder may only get 1–3 measurements during the season, which provides a limited amount of information for making selection decisions. Because the Avenger platform can collect data at a faster rate than manual methods, more measurements over the season are possible; therefore, breeders can identify which phenotypes at what time are the most predictive for yield and select lines based on that criteria. For upland cotton grown under water-limited conditions, canopy temperature collected on DOY211 showed a significant correlation with yield, but canopy temperature taken 2 weeks earlier was not significantly correlated (Fig. 6). If a breeder only had DOY183 canopy temperature, they would only have selected three of the top 10 yielding lines, whereas DOY211 data identified 6 (Table 2). This is an example of how FB-HTPP can enhance genetic gain in a breeding program by improving the selection accuracy.

**Table 2:** The ten upland cotton breeding lines identified with the lowest canopy temperatures for 02-July (DOY183) and 30-July (DOY211) compared to the 10 best yielding lines in 2015 at the U. of Arizona Maricopa Agricultural Center in Maricopa Arizona, USA

DOY183	DOY211	Best yield
0043-28-1	0043-28-1	Ark 0701-17
0045-14-5	Ark 0701-17	Ark 0705-46
Acala 1517-08	Ark 0711-2	DP1044
Ark 0705-46	GA2011004	GA2011004
Ark 0707-33	GA2009037	GA2009037
GA2010102	GA2010074	GA2010074
GA2011124	LA12306017	LA12306017
GA2009037	LA12306028	LA12306028
GA2009100	NM 13W3007	SG 105
LA12306028	PD07092	ST4946

## Advantages and limitations

One of the primary advantages to the Avenger or any other high-throughput phenotyping platform is the reduction in time to acquire trait data compared to collecting data by hand. In 2015, the time to complete an Avenger collection was compared to the time it took a summer student, with previous experience, to collect plant height and canopy temperature in a 2.7 ha field containing 240 experimental plots of upland cotton grown under well-watered and water-limited conditions. It took the student 4 times longer to complete the data collection task, yet the tractor collected 80 times more measurements (Table 3). For dynamic traits like canopy temperature, the time it takes to complete a collection is very important, because time has a very large impact on the trait itself. The average canopy temperature for each of the 240 experimental plots collected by the Avenger platform and the student on 30-July 2015 is shown in Additional file 3: Figure S3. The student started collecting at 09:00 in the north-western most plot and finished at approximately 13:00 (MST) in the south-eastern most plot. The tractor followed the same path but started at 11:00 and finished at approximately 12:30 (MST). The effect of time of day on canopy temperature is evident in the data collected by the student, because canopy temperature measurements increased following the student’s path through the field, indicating the measurements were confounded by increases in air temperature. With this data the irrigation treatments are not apparent as they are in the data collected by the tractor, which could influence a breeder’s decision on which lines to keep for the following year.

**Table 3:** A comparison of collecting plant phenotypes in the field between the Avenger tractor and 1 well-trained summer student

	Avenger tractor	Summer student
Total collection time (h)	1.5	4.0
Plots sampled per hour	110	60
Measurements per plot	120	3
Traits per plot (simultaneous)	4	2
Total measurements collected	115,200	1440
Time to upload/process data (h)	6	2.5

These values are averaged over 6 collections from a 2.7 ha field with 240 experimental plots (12 × 1.02 m) over 6 irrigation basins

For non-dynamic traits such as plant height, the time to collect the data is not as influential; however, because data is captured at such a high rate, in-plot variation can be detected and explored. The average plant height for each of the 240 experimental plots collected by the Avenger platform and the student on 16 July 2015 (DOY197) 2015 is shown in Additional file 4: Figure S4. Comparing the Avenger data to the manual measurements showed a root mean squared error (RMSE) of 6.7 cm. The within plot variation detected by the three manual measurements per plot ranged from 0.0 to 13.1 cm with an average standard deviation from the mean of 3.2 cm. The within plot variation detected by the 120 Avenger measurements per plot ranged from 7.2 to 12.3 cm with an average standard deviation from the mean of 4.3 cm. Since the lines in this trial were advanced breeding lines, little to no segregation of traits was expected, and the larger range of height variation in the manual measurements was likely due to human error. The ability to detect within-plot variation with confidence will be especially important in early-generation field trials where segregation of traits is expected to occur. Due to the large amount of data collected by the Avenger platform, that variation can be quantified and used to select or discard lines for the following year.

The time to upload and process data from the Avenger platform takes more than double that of hand measurements. This is due primarily to the double transfer of data, from the PXIe to the hard drive, then the hard drive to the server. This limitation is due to local network security. If the server were not on the local network only a one step data transfer would be needed which would reduce the time by ~ 2 h making the total time to collect and process data ~ 3.5 h rather than 7.5 h. The large volume of data collected by the Avenger or any other high-throughput phenotyping platform could also be a limitation for some breeders. The initial cost for the server and JBODS that store the data, database, and processing pipeline was ~ $49,500 USD while the tape back-up system was ~ $12,000 USD. The annual cost in maintenance, upkeep, and new tapes is ~ $3,000 USD. This annual cost does not include the salary of the information technology (IT) specialist who oversees the maintenance and system back-ups. Breeders that have a limited research budget may need to explore alternatives, such as cloud-based storage and data processing. The cost of the Avenger platform itself may also be prohibitive to many breeders. The high-clearance tractor was ~ $150,000 USD and the sensing and data acquisition equipment was ~ $125,500 USD (Table 1). As an inexpensive alternative, low-cost field carts have been developed [9, 11, 13, 14]. The development of custom high-throughput phenotyping platforms and processing pipelines required the cooperation of mechanical, electrical, and agricultural engineers, as well as IT specialists and data scientists. Institutions that do not have access to these groups of people may need to explore other, more turn-key, options.

Some final limitations of the Avenger platform or any large terrestrial based platform is field accessibility. The platform cannot enter a field after heavy rainfall because of the potential to get the platform stuck or cause soil compaction and ruts. Smaller terrestrial platforms, such as proximal sensing carts, have less wet soil restrictions because they are lighter weight and have smaller wheels. Gantry style or cable driven systems, and unmanned aerial systems (UAS) have no field accessibility concerns although wind speed or airspace regulations in the case of UAS may present difficulties for some field areas. The robustness of the Avenger or tractor-based platforms makes it possible to mount more sensors which may not be possible on smaller, lighter framed carts. However, image-based sensors (ie. hyperspectral, RGB, or chlorophyll fluorescence cameras) can require nearly 10–20 × the number of images to cover a field when compared to an UAS or gantry system because of the proximity to plants. For high-throughput plant phenotyping to be effective, users need to identify what sensors are most appropriate for their objectives and the best way to deploy those sensors.

## Conclusions

The modified Avenger-Pro high-clearance terrestrial phenotyping platform is a robust field-based, high-throughput plant phenotyping platform capable of collecting high quality data in a relatively short period of time. The semi-automated processing pipeline and database developed for the platform, cleaned, organized, stored in a database, and provided data visualization in a relatively short period of time. The database, and user supports tools (ie. Jupyter Notebooks) are easy to use and access which provides opportunities for data sharing and collaboration of these large datasets. The database and associated metadata provide the means to curate and maintain these large datasets for future use in crop modeling or machine learning algorithms. The versatile workflow and data tools presented in this paper can be applied to other FB-HTPP platforms with minimal effort.

## Supplementary information

**Additional file 1: Figure S1** The Avenger-Pro high-clearance tractor fitted with a modified front boom that carried the proximal sensor array. Each number corresponds to the “Figure No.” column in Table 1, which provides a description of the equipment, the approximate cost, and purpose.

**Additional file 2: Figure S2** Avenger tractor lead technician, Matthew Conley, performing pre-collection warm up over the white calibration panel, setting the boom height, and double-checking data loggers in the field. An example of transcribed metadata.

**Additional file 3: Figure S3** Plot-level means for canopy temperature among 33 upland cotton lines grown under well-watered and water limited conditions on 30 July 2015 (DOY211) at the U. of Arizona, Maricopa Agricultural Center in Maricopa Arizona, USA from the Avenger platform (left) and manual student collection (right).

**Additional file 4: Figure S4** The plant height plot level means for 33 upland cotton lines grown under well-watered and water limited conditions collected on 16 July 2015 (DOY197) at the U. of Arizona Maricopa Agricultural Center in Maricopa Arizona, USA from the Avenger platform (left) and manual student collection (right).

**Additional file 5: Table S1** Startup procedure.

## Data Availability

The source code for the processing pipeline, Jupyter Notebooks, and database are posted in the ALARC HTP gitlab page, access can be granted if requested. The data from the Avenger tractor can be made available upon reasonable request and by mailing us an external drive.
