# Supplementary figures and images for: A data workflow to support plant breeding decisions from a terrestrial field-based high-throughput plant phenotyping system
Source: Plant Methods. 2020 Jul 16;16:97. doi: 10.1186/s13007-020-00639-9 (PMC7364621; doi:10.1186/s13007-020-00639-9)

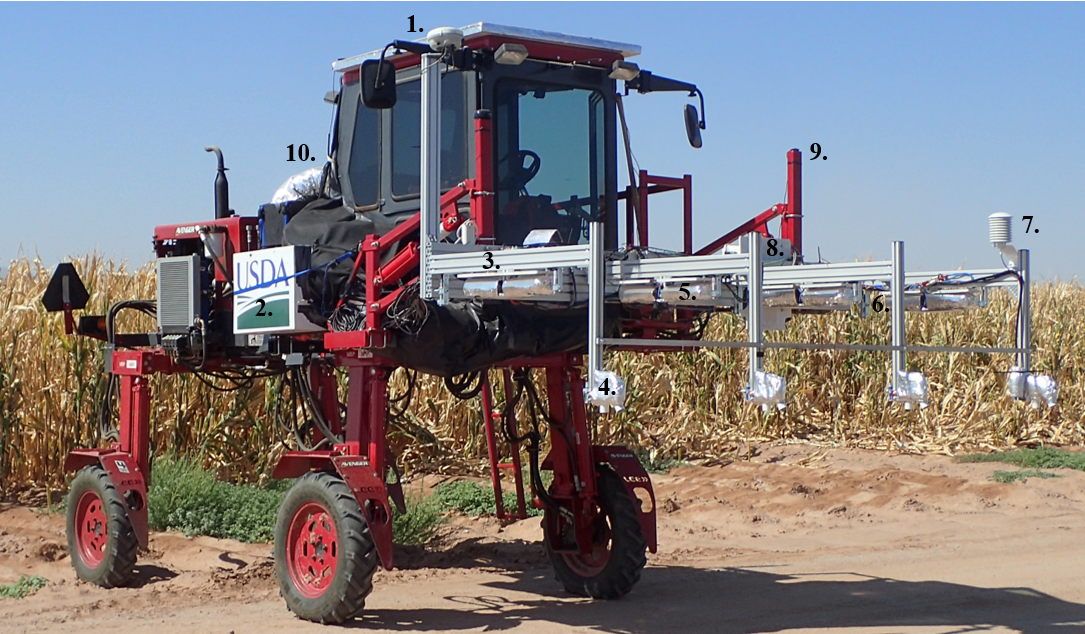

Supplement: Supplementary file 1 — Additional file 1: Figure S1 The Avenger-Pro high-clearance tractor fitted with a modified front boom that carried the proximal sensor array. Each number corresponds to the “Figure No.” column in Table 1, which provides a description of the equipment, the approximate cost, and purpose. [file 13007_2020_639_MOESM1_ESM.png]

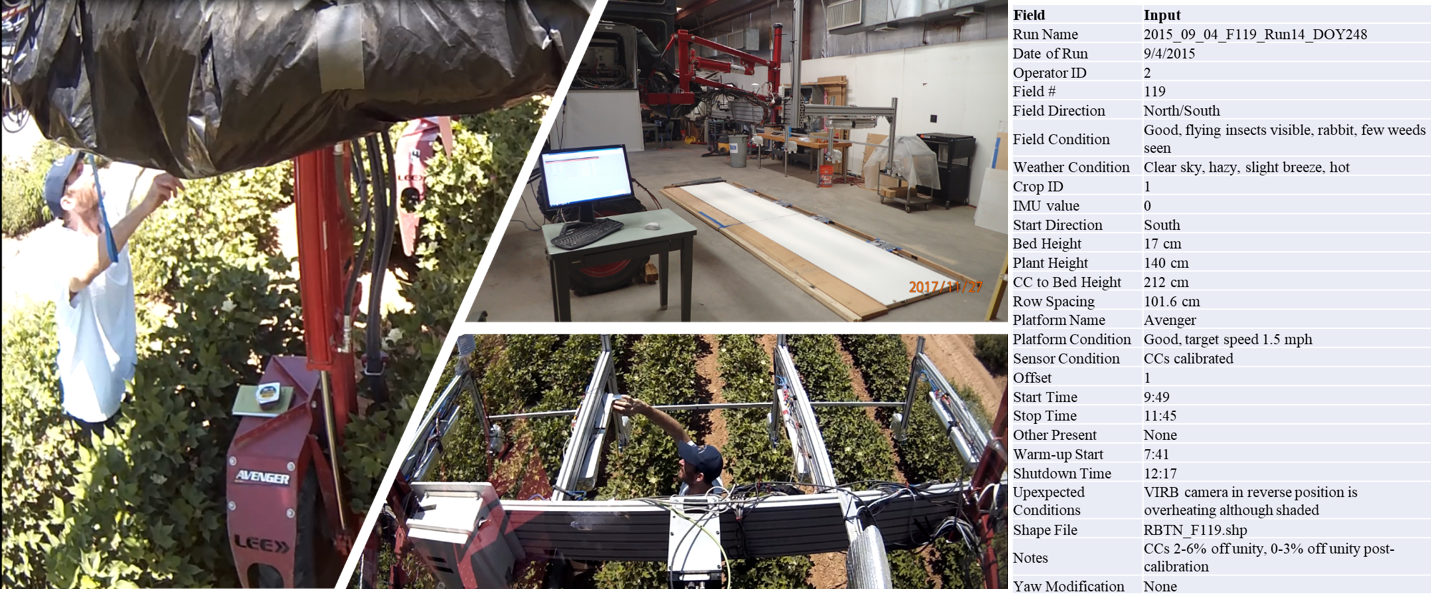

Supplement: Supplementary file 2 — Additional file 2: Figure S2 Avenger tractor lead technician, Matthew Conley, performing pre-collection warm up over the white calibration panel, setting the boom height, and double-checking data loggers in the field. An example of transcribed metadata. [file 13007_2020_639_MOESM2_ESM.png]

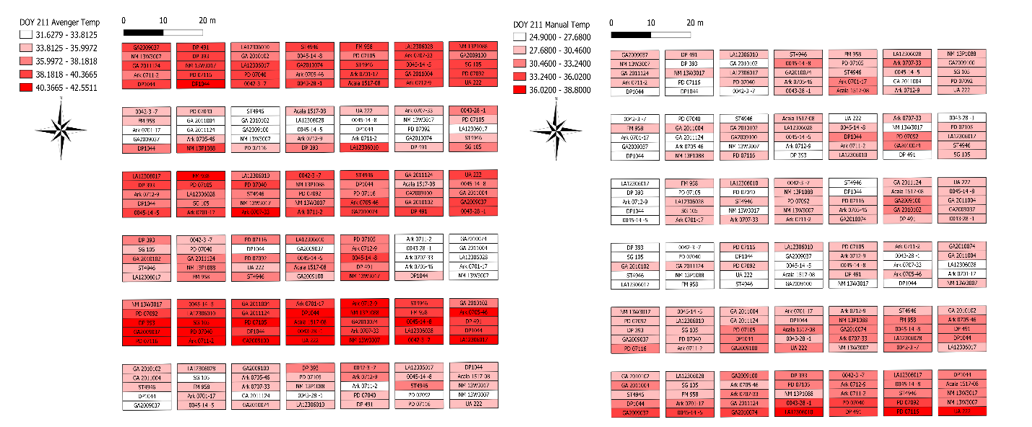

Supplement: Supplementary file 3 — Additional file 3: Figure S3 Plot-level means for canopy temperature among 33 upland cotton lines grown under well-watered and water limited conditions on 30 July 2015 (DOY211) at the U. of Arizona, Maricopa Agricultural Center in Maricopa Arizona, USA from the Avenger platform (left) and manual student collection (right). [file 13007_2020_639_MOESM3_ESM.png]

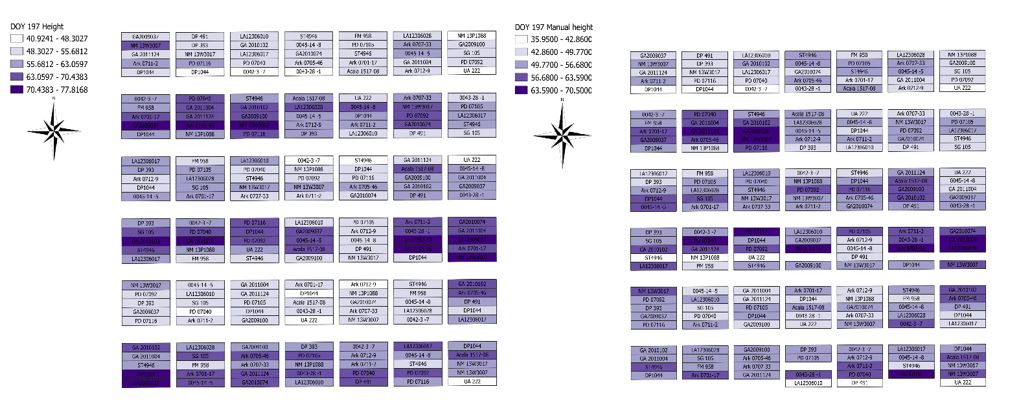

Supplement: Supplementary file 4 — Additional file 4: Figure S4 The plant height plot level means for 33 upland cotton lines grown under well-watered and water limited conditions collected on 16 July 2015 (DOY197) at the U. of Arizona Maricopa Agricultural Center in Maricopa Arizona, USA from the Avenger platform (left) and manual student collection (right). [file 13007_2020_639_MOESM4_ESM.png]
