# Supplementary material for: A data workflow to support plant breeding decisions from a terrestrial field-based high-throughput plant phenotyping system
Source: Plant Methods. 2020 Jul 16;16:97. doi: 10.1186/s13007-020-00639-9 (PMC7364621; doi:10.1186/s13007-020-00639-9)
Supplement: Supplementary file 5 — Additional file 5: Table S1 Startup procedure. [file 13007_2020_639_MOESM5_ESM.docx]

Startup procedure:

1. Visual safety and integrity check of the equipment
2. Check fuel level, oil level and hoses.
3. Remove sensor covers
4. Turn on 12v power for CropCircles and GPS
5. Turn on the GPS at unit
6. Turn on 24v power for Ultrasonic sensors
7. Check IMU USB power plug
8. Turn on PXIe at its front panel (plug in cab USB3 video screen, leave cover open until A/C)
9. [Run panel calibration of CropCircles]
10. Plug in PXIe overhead interface screen and switch on Bluetooth keyboard
11. Check sensor returns in real-time
12. Set Ipad mini secondary display
13. Turn tractor key to “on” position and wait for glow plug indicator light to go dark, then start
14. Set cab overhead A/C level
15. Start APU via small screen
16. Turn on AUX power switch to start equipment A/C
17. Turn on AUX power switch to power the receptacle extension
18. Disconnect shore power to the UPS
19. Connect the UPS to the AUX power receptacle extension
20. Verify equipment A/C operation and close PXIe front cover
21. Press and release operator toggle switch
22. Pull out tractor drive controller button
23. Flip parking break toggle switch
24. Drive
